# Nutritional Challenges in Metabolic Syndrome

**DOI:** 10.3390/jcm8091301

**Published:** 2019-08-24

**Authors:** Irene Hoyas, Miguel Leon-Sanz

**Affiliations:** 1 Department of Endocrinology and Nutrition, University Hospital Doce de Octubre, 28041 Madrid, Spain; 2 Medical School, University Complutense, 28040 Madrid, Spain

**Keywords:** metabolic syndrome, insulin resistance, dietary pattern, carbohydrates, fat

## Abstract

Metabolic Syndrome (MetS) is a combination of risk factors for the development of cardiovascular disease (CVD) and type 2 diabetes. Different diagnostic criteria were proposed, but a consensus was reached in 2009 based on values of waist circumference, blood pressure, fasting glycemia, triglycerides, and high-density lipoprotein (HDL)-cholesterol levels. The main underlying etiologic factor is insulin resistance. The quality and quantity of individual macronutrients have an influence on the development and resolution of this syndrome. However, the main treatment goal is weight loss and a decrease in insulin resistance. A controlled energy dietary recommendation, together with moderate levels of physical activity, may positively change the parameters of MetS. However, there is no single dietary or exercise prescription that works for all patients. Dietary patterns such as Mediterranean-style, dietary approaches to stop hypertension (DASH), low-carbohydrate, and low-fat diets can ameliorate insulin resistance and MetS. Long-term adherence to a healthy lifestyle is key in assuring that individuals significantly reduce the risk of CVD and diabetes mellitus.

## 1. Introduction

Metabolic Syndrome (MetS) is a combination of risk factors for the development of cardiovascular disease (CVD) and type 2 diabetes (T2DM). It is very common and occurs in all regions of the world in populations with reduced physical activity and excessive energy intake. Age, sex, socioeconomic status, and ethnic background may change the prevalence, but it is estimated that 25–35% of adults may have MetS. The grouping of risk factors for CVD was firstly proposed 100 years ago but was progressively developed over many decades and received different names, such as syndrome X, insulin resistance syndrome, and the deadly quartet [1].

MetS was also defined in various ways over time by different organizations and expert groups. There are four common components present in the different definitions: obesity, abdominal adiposity or indicators of insulin resistance, impaired glucose metabolism, hypertension, and atherogenic dyslipidemia. The differences among the diverse definitions depend on the cut-off points needed to fulfil the diagnostic criteria and the requirement of the obligatory presence of specific features to meet the definition of MetS [2].

In 2009, six organizations, the International Diabetes Federation, the American Heart Association, the National Heart, Liver, and Blood Institute, the World Heart Federation, the International Atherosclerosis Society, and the International Association for the Study of Obesity, reached a consensus for the definition of MetS [3]. According to the consensus, MetS can be diagnosed if the patient has any three of the following:

▪ Elevated waist circumference: population- and country-specific cut-off points;
▪ Blood pressure: systolic > 130 and/or diastolic > 85 mmHg or drug treatment;
▪ Fasting glucose: >100 mg/dL (5.6 mmol/L) or drug treatment;
▪ Triglycerides: >150 mg/dL (1.7 mmol/L) or drug treatment;
▪ High-density lipoprotein (HDL)-cholesterol: <40 mg/dL (1 mmol/L) (male) or <50 mg/dL (1.3 mmol/L) (female) or drug treatment.

MetS is not a disease but a cluster of individual risk factors, whose main purpose is to identify individuals with increased risk of developing CVD and diabetes mellitus. MetS facilitates the early identification of patients with excessive adipose tissue and insulin resistance, despite the fact that not every person at risk who fulfils the criteria for MetS has insulin resistance and, on the contrary, not all persons with insulin resistance have MetS [4]. The diagnosis of MetS may be helpful in convincing patients about the importance of adopting therapeutic measures to correct the different components of this syndrome. Patients who fulfil the criteria of MetS have a two-fold increased risk of CVD, 1.5-fold increased risk of all-cause mortality, and three-fold increased risk of diabetes [5,6]. These estimates may differ slightly depending on the MetS criteria set used and the population where they are applied.

However, the definition of this syndrome does not include some relevant CVD risk factors, such as family history, smoking habit, age, gender, or low-density lipoprotein (LDL)-cholesterol. That may explain why MetS is a good prognostic estimation in the long term, but other risk calculators may be more precise for prognosis at less than 10 years. MetS could also indicate risks not accounted for in other CVD risk calculators. In certain instances, it can move the CVD risk of a patient upward, from low to intermediate risk, according to traditional risk calculators, such as ATP-III, SCORE, or Framingham risk score [7].

## 2. Therapeutic Approach to Metabolic Syndrome

Among other reasons, sedentary life and the easy access to inexpensive foods contribute to the explanation of why MetS is currently so prevalent. Its treatment aims to decrease the risks of CVD and T2DM. The first and most important step is the implementation of a new lifestyle with changes in diet and physical activity, as well as the acquisition of healthier habits. Weight loss and lifestyle changes may improve individual MetS components. Behavioral interventions make it easier for individuals to incorporate and maintain these changes in their daily routines. 

Weight reduction was the main goal of most intervention studies. It is associated with significant improvements in all parameters of MetS. Even moderate weight loss (around 7%) resulted in substantial reductions in blood pressure, and glucose, triglyceride, and total cholesterol concentrations [8,9]. In addition, weight reduction improves adipokines and inflammation markers, such as adiponectin and tumor necrosis factor alpha concentrations [10].

A reasonable first goal for obese patients is to aim for weight loss of approximately 10% of baseline weight in six months. If they achieve this objective, insulin resistance will improve along with risk reduction of MetS and CVD. Even a lower weight loss, between 5% and 10%, improves the sensitivity to insulin between 30 and 60%, an effect greater than that seen with insulin-sensitizing drugs [11].

Considering all the therapeutic options, caloric restriction is a very effective intervention because most persons with this syndrome are obese and sedentary. Changes in physical activity are always part of lifestyle interventions for MetS, and current scientific evidence supports the role of exercise as an effective treatment strategy for the syndrome. Along with dietary changes, a program of regular physical activity also leads to a reduction in insulin resistance and CVD risk [12].

In this review, we focus on the nutritional component of MetS therapy. We analyze the contributions of the three macronutrients to the development and treatment of MetS. We do not cover issues related to micronutrients, sweeteners, or alcohol consumption.

## 3. Influence of Dietary Macronutrients in MetS

The role of each macronutrient present in the diet in the development and treatment of MetS was extensively studied and we review the current state of knowledge of their contributions to this syndrome.

### 3.1. Carbohydrates

After oral administration, absorption, gastrointestinal and pancreatic hormone secretion, liver metabolism, and visceral and muscular uptake, carbohydrates are responsible for blood glucose and insulin levels. They should not be regarded as a homogeneous component of foods. Overall, there are three types: starch or complex carbohydrates, sugars or simple carbohydrates, and fiber, with different repercussions on the glycemic response. The metabolic effects of foods containing carbohydrates can be partially predicted by their glycemic index (GI), which ranks foods containing carbohydrates according to how they change blood glucose levels, usually by comparing 50 g of any given food with a reference food such as white bread. When the GI is low (55 or less) that particular food causes a lower and slower increase in blood glucose and, therefore, insulin levels. Related to this index is the concept of glycemic load (GL), which combines both the quantity and quality of carbohydrates. If the carbohydrate content of 100 g of potato is 14 g and the GI is 85, the GL will be 85 × 14/100 = 12, more than double the GL of 100 g of an apple, which is 5. The GL concept allows comparison of blood glucose response to different types and amounts of foods [13,14]. The GI depends not only on the carbohydrate composition but also on other factors (the physical form of the food, amylose or amylopectin content, complete composition of the food, presence of fiber, cooking process, etc.) [15]. Despite its conceptual appeal, the utility of GI and GL in clinical practice is not widely recognized because of lack of impact on glycemic control [16].

It is accepted that an elevated intake of carbohydrates of high GI causes insulin resistance directly and contributes to the development of T2DM in persons with MetS [17,18]. Generally, foods with low GI are also more abundant in fiber. A diet rich in fiber showed a reduction in insulinemia by 10% and insulin resistance (by HOMA index) by 13% [19]. Fiber intake increases satiety and also reduces the risk of developing T2DM, and, in patients already diagnosed with this disease, viscous fiber supplements improve conventional markers of glycemic control beyond usual care and should be considered in its management [20]. However, fiber intake through foods that are naturally high in dietary fiber is better than fiber supplements, because natural foods also provide other substances such as micronutrients and phytochemicals.

Carbohydrate and lipid contents of diets have a mutual influence on their metabolism. Diets with low GI produce lower concentrations of fasting triglycerides and LDL-cholesterol [21]. It is interesting to emphasize that, when simple carbohydrates are consumed in a proportion lower than 20–25%, they do not modify the levels of plasma triglycerides. However, in obese patients with insulin resistance, the intake of simple carbohydrates stimulates the synthesis of fatty acids and inhibits endothelial and hepatic lipoprotein lipase, and in this way promotes hypertriglyceridemia and lowers HDL-cholesterol levels [22]. In a more positive manner, the effect of carbohydrates on the increase in plasma triglyceride levels is lower if fiber intake is high. Low-glycemic-index diets are, thus, recommended for patients with MetS [23,24], contributing to decreased CVD risk, and reduced levels of glycosylated hemoglobin in type 1 and 2 DM patients [25,26].

In the last two decades, we saw a growing interest in diets with low carbohydrate content or ketogenic diets. Initially, they were discredited because it was thought that the elevated lipid content would increase the risk of CVD. However, several landmark studies showed greater weight loss during the first six months of follow-up compared to conventional low-fat hypocaloric diets [27,28]. No statistically significant differences in weight loss were seen after 12 months of follow-up. Interestingly, with these ketogenic diets, triglyceride concentrations decrease, HDL-cholesterol levels increase markedly, and insulin sensitivity and glycosylated hemoglobin (HbA1c) improve. In those cases of high HbA1c, there is a greater reduction in HbA1c levels after carbohydrate restriction rather than after protein or lipid restriction [29].

Fructose is a simple carbohydrate that deserves particular comment. When absorbed, it does not stimulate the secretion of insulin or leptin. Long-term daily consumption leads to an increase in weight and a decrease in insulin sensitivity, favoring the development of MetS and type 2 diabetes [30], both in children and in adults [31]. Soft drinks contain large amounts of high-fructose corn syrup (HFCS) and sucrose and add greater amounts of simple carbohydrates to the daily diet. It was stated that moderate fructose consumption of ≤50 g/day or ~10% of energy has no deleterious effect on lipid and glucose control, and ≤100 g/day does not influence body weight. Nevertheless, a higher intake is not recommended, and the intake of sugar-sweetened beverages should be limited or avoided [32].

### 3.2. Lipids

In humans, energy intake is made up of carbohydrates and lipids. However, lipids also have functional features and play an important role in the pathogenesis of atherosclerosis. The accepted range of lipid calories in the diet is very broad and is the opposite of carbohydrates. Therefore, low-lipid or low-carbohydrate diets contain very different total amounts of lipids. For all adults, the acceptable macronutrient distribution of total fat is to be 20–35% of total calorie intake [33].

The amount of fat can influence insulin sensitivity and the risk of developing type 2 diabetes only with intakes greater than 35–40% of total energy intake [34]. A diet that contains 20–40% fat does not change insulin sensitivity, regardless of its effect on weight [35].

However, lipids in the diet are a heterogeneous group, and quality is as important as quantity. Generally, we classify lipids as saturated, monounsaturated, and polyunsaturated fats (SFAs, MUFAs, and PUFAs, respectively). The consumption of high amounts of saturated fats and *trans* fatty acids is associated with an alteration in the action of insulin, while the intake of monounsaturated fats has the opposite effect. Therefore, the ratio of monounsaturated fatty acids/saturated fat is related to insulin sensitivity (Table 1). Along with these effects on insulin, diets enriched with MUFAs improve the lipid profile, because they reduce LDL-cholesterol and triglycerides, and elevate HDL-cholesterol levels [36,37,38]. Polyunsaturated fats are associated with a lower relative risk of 40% for developing type 2 diabetes. In studies that included patients with type 2 diabetes, substitution of SFAs by PUFAs and carbohydrates by MUFAs caused a decrease in insulin resistance. Moreover, ω-3 PUFAs can reduce triglyceride levels, improve hypertension, reduce inflammation, and diminish cardiovascular risk in diabetic patients [39,40,41,42]. It was recently reported that the intake of 2 g of icosapent ethyl twice daily with statin therapy is associated with less CVD morbidity and mortality but with slightly higher rates of hospitalization for atrial fibrillation and serious bleeding [43].

A diet low in fat and rich in simple carbohydrates, as was used in the CARMEN study, may increase insulin resistance (measured by HOMA) and is associated with a significant increase in triglyceride levels [44]. For some time, the American Diabetic Association (ADA) recommended that the sum of carbohydrates and monounsaturated fatty acids should represent 60–70% of the total energy in the diet. Nevertheless, since the ADA 2014 position statement, there is no “first-line” approach with respect to the optimal carbohydrate quantity in the diet plan, because evidence remains inconclusive [45]. On the other hand, in overweight or obese individuals, low-fat diets are equal to but no better than other weight-reducing diets when the goal is weight reduction [46].

A healthy pattern limits saturated and *trans* fats, added sugars, and sodium. The recommendation for the general population is to consume less than 10% of calories per day as added sugars and less than 10% of calories per day as saturated fats. In the Dietary Guidelines for Americans, it is advised that individuals eat as little dietary cholesterol as possible while consuming a healthy eating pattern. The cholesterol intake in a healthy United States (US)-style diet contains approximately 100 to 300 mg of cholesterol across the 12 calorie levels [47]. European guidelines recommend that foods rich in *trans* or saturated fats (hard margarines, tropical oils, fatty or processed meat, sweets, cream, butter, and regular cheese) should be replaced with monounsaturated fats (extra virgin olive oil) and polyunsaturated fats (non-tropical vegetable oils). In this way, it is assumed that *trans* fats will be <1.0% of total energy and saturated fat <10% (<7% in the presence of high plasma cholesterol values) [48].

### 3.3. Proteins

Proteins are associated with increased satiety and the preservation of lean body mass during weight loss, but their role in the dietary recommendations for patients with MetS is less clear.

Guidelines recommend a wide range of 10–35% of energy intake as digestible protein for adults, or a minimum of 0.8 g/kg body weight per day. Within this range, ADA position statements suggest that patients with diabetes and normal renal function should consume 15–20% of their energy intake as protein. However, they recognize that there is no definitive evidence for recommending an ideal amount of protein in relation either to glycemic control or for improving CVD risk factors [49].

Protein may have an incretin role. Its consumption is associated with higher insulin secretion, equivalent to that caused by eating the same amount of glucose. Some amino acids, such as leucine, lysine, or alanine, stimulate insulin secretion. In contrast, homocysteine can inhibit it [50,51].

There are several studies that propose hyperproteic diets in the management of MetS due to the satiating effect of proteins [52,53,54]. These diets also contribute to the preservation of lean mass. However, these diets may favor an increase in urinary calcium excretion and bone remodeling, and their use is not totally accepted [55]. Overall, these concerns seem a little overstated. High-protein diets do not seem to lead to calcium bone loss, and have no damaging effect on the kidney unless there is a pre-existing metabolic renal dysfunction.

## 4. Dietary Patterns

The most effective intervention for metabolic intervention is caloric restriction. Nutrition change to support a 7–10% weight loss is an appropriate goal for people with prediabetes, unless additional weight loss is desired for other purposes. The contributions of different nutrients to success in the reduction have to be seen in the context of the general eating plan of the patient. There is no perfect combination of macronutrients useful for all individuals. Compliance with a healthier lifestyle and dietary intake are more important than a particular dietary pattern. This represents an advantage for patients confronting MetS. As there is no “one-size-fits-all” pattern, individuals can advance with any healthy plan that is easy for them to follow. It also opens the door to adaptations of dietary recommendations based on metabolic goals, socioeconomic factors, food availability, and personal and cultural preferences. Irrespective of the macronutrient balance in the diet, total energy intake should be appropriate to accomplish the weight management goals.

Several nutrition patterns are effective in improving diabetes control, but the optimal macronutrient composition in meal planning in persons with MetS is less well defined. As previously seen, the main concern with regard to MetS is the development of diabetes and the associated cardiovascular risk. Patients with MetS have a similar benefit to overweight/obese patients with diabetes from the adoption of several dietary patterns. As already stated, the goal in both conditions is achieving weight loss. A short description of the characteristics of the different dietary patterns and their potential benefits was recently published [49]. Multiple studies analyzed the effectiveness of different patterns and confirmed the emphasis on compliance. Table 2 summarizes the main benefits of different dietary patterns regarding weight, glucose and lipid metabolism, and blood pressure.

With the exception of low-carbohydrate diets, common to many of these patterns is the emphasis on no starchy vegetables, the reduction of added sugars and refined grains, and the exclusion of processed foods to favor whole foods instead. Adoption of a Mediterranean-style diet rich in whole grain cereals, fruits, vegetables, nuts, and olive oil, compared to a prudent dietary pattern (50–60% of energy as carbohydrate and <30% as fat), was associated with improvements in endothelial function and significant reductions in the markers of systemic vascular inflammation in MetS patients after two years of follow-up [60,61]. The DASH (dietary approaches to stop hypertension) diet, rich in fruits, vegetables, and low-fat dairy food and low in saturated and total fat intake, demonstrated weight reduction and a significant reduction in blood pressure [62,63,64]. Mediterranean-style, vegetarian, and DASH eating patterns have a lower risk of developing type 2 diabetes [59]. Overall, the lower the provision of carbohydrates is, the lower the value of A1c hemoglobin is [65]. Weight loss or A1c reductions may be statistically significant but with small differences, with, for example, vegetarian diets, of 2 kg and 0.3%, respectively [66].

Low-fat diets are used as the control or default intervention vs. other dietary patterns. Their benefits seem to derive from weight loss rather than the eating pattern itself [67]. Interestingly, when low-carbohydrate diets are used, the specific distribution of fats and, particularly, the amount of saturated fat must be taken into account because, otherwise, it may be higher than that recommended for healthy individuals [68].

If we can call intermittent fasting a dietary pattern, different forms of intermittent fasting can lead to improved beta cell responsiveness, insulin sensitivity, and blood pressure control [69].

## 5. Conclusions

MetS is a cluster of risk factors that identifies patients at risk of developing diabetes mellitus and CVD. Nutrition therapy, as part of a comprehensive lifestyle intervention, may improve obesity and insulin resistance, which play key roles in its pathogenesis. 

Macronutrients may contribute to worsening or improving MetS. An elevated intake of carbohydrates of high GI causes insulin resistance directly and has an impact on the development of T2DM in persons with MetS. In contrast, low-GI diets, more abundant in fiber, increase satiety and decrease insulin resistance and the risk of developing T2DM. Low-GI diets are, thus, recommended for patients with MetS. Diets enriched with MUFAs also improve the lipid profile and increase insulin sensitivity compared with SFAs. A healthy pattern limits saturated and *trans* fats, added sugars, and sodium. Specifically, the recommendation for the general population is to consume less than 10% of calories per day as added sugars and less than 10% of calories per day as saturated fats. Proteins in the diet are associated with increased satiety, insulin secretion, and preservation of lean body mass during weight loss, and hyperproteic diets are suggested for the management of MetS.

The effects of macronutrients are important; however, we consume them combined in eating patterns. Several dietary patterns may be helpful in reversing MetS, such as Mediterranean-style, vegetarian, DASH, low-carbohydrate, or even low-fat diets. The different patterns have variable effects on each risk factor, but all of them must be compatible with caloric restriction, which is the most effective intervention for metabolic intervention.

## Figures and Tables

**Table 1:** Influence of diet on insulin sensitivity.

Diet Component	Insulin Sensitivity
Total fatty acids (>40%)	(−)
Polyunsaturated fatty acids	(−)
*trans* Fatty acids	(−)
Monounsaturated fatty acids	(+)
Fiber cereal	(+)
Low glycemic index	(+)
Alcohol	(+)
Salt	(−)
Simple sugars (>20% energy)	(−)
Conjugated linoleic acid	(−)

(+): Increases insulin sensitivity. (−): Decreases insulin sensitivity.

**Table 2:** Metabolic syndrome improvement observed with different dietary patterns. DASH—Dietary approaches to stop hypertension; CVD—Cardiovascular disease; LDL-C—Low-density lipoprotein cholesterol; HDL-C—High-density lipoprotein cholesterol.

Dietary Patterns	Main Reported Benefits						
	↓ Risk of Diabetes	↓ A1c	↓ Triglycerides	↓ CVD	Weight Loss	↓ LDL-C or HDL-C	↓ Blood Pressure
Low fat ^1^	X				X		
Very low fat ^2^					X		X
Low carbohydrate ^3^		X			X	X	X
Very low carbohydrate ^4^		X	X		X	X	X
Mediterranean-style	X	X	X	X			
DASH	X				X		X
Vegetarian or vegan	X	X			X	X	
Paleo diet	Lack of evidence						
Zone diet (40–30–30 diet)	Lack of evidence				x	Lack of evidence	
Healthy Nordic diet	x			x	x		

The content of the table is derived from References [56,57,58,59]. X means there is proven evidence of the benefit; x indicates hypothetical evidence. “↓” represents a decrease and improvement in risk factor. ^1^ Low fat = fat intake < 30% of total calories; ^2^ very low fat = fat intake <10% of total calories; ^3^ low carbohydrate = carbohydrate intake 26–45% of total calories; ^4^ very low carbohydrate = carbohydrate intake < 26% of total calories.
